# A Paradoxical Chemoresistance and Tumor Suppressive Role of Antioxidant in Solid Cancer Cells: A Strange Case of Dr. Jekyll and Mr. Hyde

**DOI:** 10.1155/2014/209845

**Published:** 2014-04-03

**Authors:** Jolie Kiemlian Kwee

**Affiliations:** Coordenação de Pesquisa, Instituto Nacional de Câncer, Rua André Cavalcante 37, 20231-050, Rio de Janeiro, RJ, Brazil

## Abstract

Modulation of intracellular antioxidant concentration is a double-edged sword, with both sides exploited for potential therapeutic benefits. While antioxidants may hamper the efficacy of chemotherapy by scavenging reactive oxygen species and free radicals, it is also possible that antioxidants alleviate unwanted chemotherapy-induced toxicity, thus allowing for increased chemotherapy doses. Under normoxic environment, antioxidants neutralize toxic oxidants, such as reactive oxygen species (ROS), maintaining them within narrow boundaries level. This redox balance is achieved by various scavenging systems such as enzymatic system (e.g., superoxide dismutases, catalase, and peroxiredoxins), nonenzymatic systems (e.g., glutathione, cysteine, and thioredoxin), and metal-binding proteins (e.g., ferritin, metallothionein, and ceruloplasmin) that sequester prooxidant metals inhibiting their participation in redox reactions. On the other hand, therapeutic strategies that promote oxidative stress and eventually tumor cells apoptosis have been explored based on availability of chemotherapy agents that inhibit ROS-scavenging systems. These contradictory assertions suggest that antioxidant supplementation during chemotherapy treatment can have varied outcomes depending on the tumor cellular context. Therefore, understanding the antioxidant-driven molecular pathways might be crucial to design new therapeutic strategies to fight cancer progression.

## 1. Introduction

**Figure 1:**
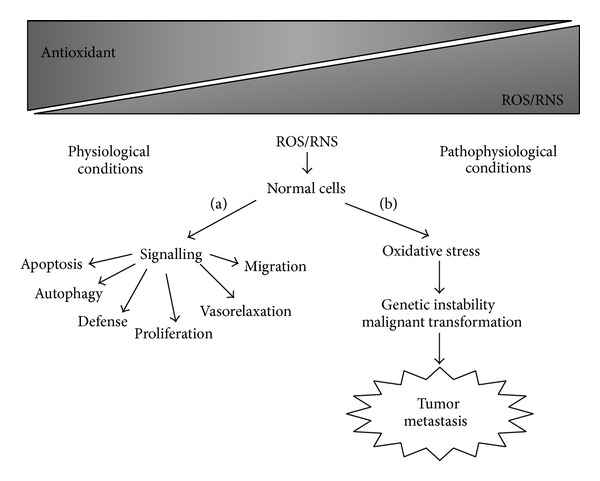
Schematic representation of reactive oxygen and nitrogen species (ROS/RNS) inductions in physiological (a) and pathophysiological (b) conditions.

**Figure 2:**
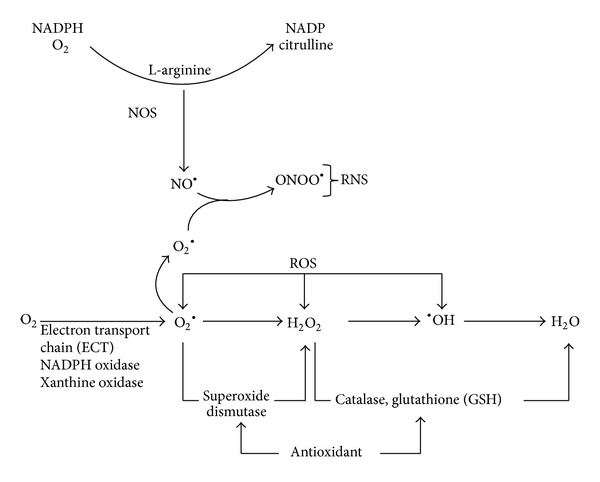
Sources of reactive oxygen (ROS) and nitrogen (RNS) species production. Enzymatic and nonenzymatic antioxidants counterbalance it.

Reactive oxygen and nitrogen species (ROS/RNS) are oxidants natural products formed during cell vital metabolism activity that orchestrate the transmission of regulatory signals for proliferation, migration, defence, vasorelaxation, autophagy, and apoptosis signals (Figure 1(a)) [1–12]. Progress in redox biochemistry study has revealed an oxygen adaptation, whereby the cell has acquired the capability to initiate changes to the local redox environment as a means of regulating signaling pathways [1–4, 6–11]. This has changed the way cellular oxidant production is viewed, from a simplistic model where all oxidant production is inherently damaging to a more complex scenario where a regulated small increase in oxidant production can be essential for optimal cellular function (Figure 1) [1–12]. In this model ROS and RNS act as second messengers, forming an integral part of the signal transduction network [1–4, 9, 11, 12]. Reactive nitrogen species are produced by the endothelium inducing vascular relaxation when vascular smooth muscle cells were stimulated with vasodilators such as acetylcholine, histamine, and bradykinin. Nitric oxide synthase catalyzes a five-electron oxidation of a guanidine nitrogen of L-arginine in the formation of citrulline and nitric oxide [7–9, 11, 12]. On the other hand, ROS are heterogeneous group diatomic oxygen derived of free and nonfree radicals species with a wide range of reactivity [10–12]. Their formation begins with the univalent reduction of oxygen to produce superoxide radical (O_2_^•−^), a free radical that gives rise to many highly reactive species such as hydroperoxyl radical (HO_2_^•^), hydrogen peroxide (H_2_O_2_), and hydroxyl radical (^•^OH) (Figure 2) [10–12]. For example, superoxide can dismutate to form hydrogen peroxide (H_2_O_2_), a membrane-permeable, mildly prooxidant molecule which in turn can lead to formation of several highly oxidizing derivatives such as hydroxyl radicals (Figure 2). Also, O_2_^•−^ can react with nitric oxide (NO^•^) resulting in peroxynitrite (OONOO^−^), a high RNS (Figure 2) [11, 12]. Mitochondria form the major powerhouse of ROS production; they are generated in association with the activity of the respiratory chain such as NADH dehydrogenase enzyme complexes in aerobic ATP production [13–15]. In addition, two classic phagocytic ROS-generating enzymes use molecular oxygen as a substrate, including the multisubunit NADPH oxidase and its homologue NOX/Duox family and myeloperoxidase in various tissues in response to extracellular influences [16, 17]. Other sources of ROS production include the cytochrome P450 (CYP450) system, which is involved mainly in removing or detoxifying toxic substances in the liver [13] and xanthine oxidase which catalyzes the oxidation of hypoxanthine to xanthine with the formation of H_2_O_2_ [18]. The imbalance of this cellular redox state is characteristic of many diseases where abnormal oxidant production causes extensive tissue damage (Figure 1(b)) [3, 6, 19]. Antioxidant has been defined as any substance that significantly delays or prevents oxidative damage of an oxidizable substrate (Figure 2). Due to their high reactivity, the ROS production levels are tightly controlled by antioxidants to avoid oxidative stress and, eventually, oxidative damage which is frequently linked to genetic instability, tumor promotion, and metastasis (Figure 1) [20]. On the other hand, the primary mechanism of many chemotherapy drugs and ionizing radiation, widely used against cancer cells, is the formation of ROS [11, 21]. At this point, questions arise whether reduction of oxidative stress in tumor cell environment with antioxidant treatment would be beneficial or not [22]. Moreover, it should be stressed that the antioxidants cannot distinguish between the radicals that play a beneficial role and those that cause carcinogenesis. Understanding the biological redox system for the development of more effective and less toxic chemotherapy ROS induction strategies for cancer cells is deserved [21, 22]. Therefore, the modulation of intracellular antioxidant concentration is a double-edged sword, with both sides exploited for potential therapeutic benefits.

## 2. ROS and Hypoxia in Solid Tumors

**Figure 3:**
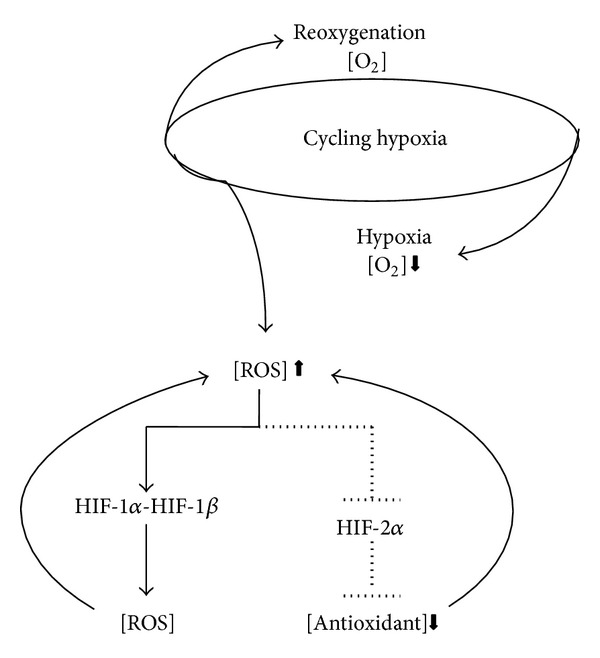
Schematic representation of cycling hypoxia effects on ROS production through activities modulations of HIF-1*α* and HIF-2*α*.

**Figure 4:**
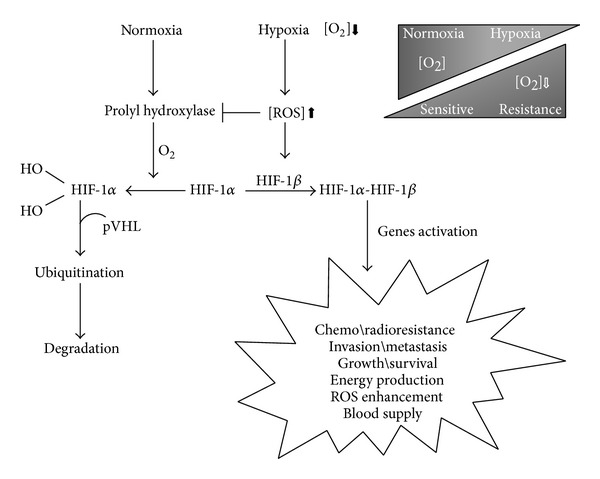
Role of ROS in hypoxia and normoxia.

Solid tumors are known to have a poor microvascular network and high interstitial fluid pressure resulting in hypoxic environment conferring chemo and radiotherapy resistance [22]. There are three major forms of hypoxia that varies with the duration: acute, chronic, and intermittent. Acute hypoxia occurs when tumor vessels become temporarily hypoxic for a period of seconds or a few hours. Chronic hypoxia is a progressive and severe reduction in oxygen (hours to days) [22]. Intermittent hypoxia, also referred to as cycling hypoxia, is characterized by cyclic periods of hypoxia and reoxygenation and plays the main role in resistance of solid tumor treatments (Figure 3) [23–26]. Hypoxic microenvironments are characterized by extreme heterogeneities in tumor cells oxygenation that arise as a result of the increased oxygen diffusion distance due to tumor expansion and poorly developed vascular networks [22, 27]. Gradients in oxygen are frequently found surrounding perfused vessels, ranging from normal values near the blood vessel to complete anoxia adjacent to necrosis [27, 28]. The balanced proportion of hypoxic cells in cancer is driven by the tolerance of individual cells to these different types of hypoxia and varies remarkably among different tumors with otherwise similar clinical features [29]. These differences are important, because the fraction of viable hypoxic cells is a major determinant of prognosis, as hypoxic cells are highly resistant to chemotherapy and radiation therapy (Figure 3). Reducing cellular tolerance to hypoxia is therefore a strategy to reduce the proportion of hypoxic cells in tumors to improve current cancer therapy [27–33]. Tumor cells can adapt to hypoxic conditions by employing a variety of survival tools, which result in the promotion of cancer cell growth and metastasis [22, 32]. This adaptation is mainly mediated by hypoxia-inducible factor-1 (HIF-1) (Figure 3). HIF-1 is a heterodimeric transcription factor consisting of an oxygen-regulated subunit (HIF-1*α*) and a stable nuclear factor, HIF-1*β* aryl hydrocarbon receptor nuclear translocator (ARNT). Under normoxic conditions, HIF-1*α* is hydroxylated by prolyl hydroxylase (PHD) at proline 402 and proline 564, and the hydroxylated HIF-1*α* recruits von Hippel-Lindau (pVHL), an E3 ubiquitin protein ligase, and is rapidly degraded by the proteasome after being targeted for ubiquitination (Figure 3). Under hypoxic conditions, cytosolic HIF-1*α* is stabilized by inhibition of the oxygen- and PHD-dependent enzymatic hydroxylation of proline residues and subsequently translocated to the nucleus, where it binds HIF-1*β* [30, 34, 35]. The complex binds to the hypoxia-response element in its targets, which results in the transactivation of numerous genes encoding proteins necessary for the blood supply, energy production, growth/survival, invasion/metastasis, and chemo/radioresistance (Figure 4) [30, 35]. An association of HIF-1*α* overexpression with cell proliferation and poor prognosis has been observed in many kinds of human cancers [30, 34, 35]. It is well known that hypoxic conditions increase intracellular ROS levels [14] and recent studies provide important insights into the molecular mechanisms by which cycling hypoxia increases the oxidative stress [24]. This constant generation of ROS through intensive cycling hypoxia stabilizes HIF-1*α* by preventing its degradation and induces HIF-2*α* degradation (Figure 3). Since HIF-1*α* regulates genes encoding prooxidant enzymes and HIF-2*α* is a potent regulator of the genes encoding antioxidant enzymes, it was proposed that both HIFs contribute in part to the oxidative stress caused by cycling hypoxia. [36–39]. Ironically the main mechanism of ionizing irradiation and many anticancer drugs to induce apoptosis is through ROS which activate HIF-1*α* [11, 30].

## 3. ROS and Chemotherapeutic Drugs

Despite great improvements in screening strategies and adjuvant therapies, current treatments still rely heavily on conventional chemotherapy for most cancers. Additionally, most of these conventional chemotherapies agents such as taxanes, anthracyclines, and platinum coordination complexes induce ROS [11, 40–42] and are somehow cardiotoxic [43, 44]. Hence, the efficacy of these prooxidant chemotherapeutic agents is dose-dependent, which is limited by toxicity to nontumor tissues, as a result of its poor tumor selectivity. Modulation of ROS levels by antioxidants may be effective in protecting nontumor tissues especially the heart from oxidative damage but they may also reduce the efficacy of these anticancer drugs [43]. Nevertheless, the mechanism by which these chemotherapeutic agents inducers exhibit antitumor effects is likely multifactorial. Consequently, to improve survival length and preserve quality of life, the challenge is to develop approaches aimed at increasing chemotherapy toxicity to tumor tissue while not affecting nontumor tissues [43, 44]. Therefore, the degree to which ROS contribute to the antineoplastic effects of these chemotherapeutic drugs should be evaluated.

## 4. Antioxidants Playing Hyde and Jekyll

### 4.1. Exogenous Antioxidant

In order to maintain an appropriate level of ROS and regulate their action, the body's natural defense against oxidative stress consists of several antioxidative systems. Therefore, mammalian cells have developed many enzymatic and nonenzymatic antioxidative systems [20, 45, 46] as well as transfer proteins that sequester prooxidant metals inhibiting their participation in redox reactions (Table 1) [47]. Components of the endogenous antioxidant defense system work together and in concert with dietary antioxidants (Table 2) [20, 21, 46] to prevent and reduce oxidative stress. In addition, the antioxidant activity of many of these enzymes and compounds is reliant upon minerals derived from the diet such as selenium, copper, manganese, and zinc (Table 2) [48]. Much debate has focused on the use of antioxidant supplements by patients undergoing chemotherapy due to concerns that the antioxidants may interfere with the mechanism of action of the therapeutic agent and subsequently decrease its efficacy [21, 49]. On the other hand, others argue that antioxidant supplements are beneficial to patients undergoing chemotherapy because they enhance the efficacy of the chemotherapy as well as alleviate toxic side effects, allowing patients to tolerate chemotherapy for the full course of treatment and lessen the need for dose reduction [20, 43]. Despite convincing evidence from preclinical experiments, clinical trials that tested dietary antioxidant nutrients and micronutrients as cancer chemoprevention agents have been unsuccessful or even resulted in harm [49, 50]. The lack of success in clinical trials and the discrepancies with preclinical experiments can be explained by factors, such as (i) lack of biological rationale for selecting the specific agents of interest, (ii) limited number of agents tested to date, and (iii) insufficient duration of the interventions and follow-up. Moreover, this high level of heterogeneity within epidemiological data may support the existence of other factors that could modulate the relationship between antioxidant and cancer development, explaining contrasted results across different populations [21, 49].

### 4.2. Endogenous Antioxidants

Modulation of endogenous antioxidants is among other strategies to balance the intracellular redox levels. Among the various ROS metabolically generated, H_2_O_2_, the nonradical two-electron reduction product of oxygen, emerged as central hub in redox signaling and oxidative stress. Processes such as proliferation, differentiation, inflammation, and apoptosis use H_2_O_2_ as signaling compound. Metabolic sinks of this low-molecular-weight include the peroxidatic reaction carried out by catalase and numerous peroxidases [51]. However, due to the high affinity of H_2_O_2_ for thiol residues, the new and expanding family of thiol-specific antioxidant enzymes, peroxiredoxins, has received considerable attention. Indeed, under physiological conditions, eukaryotic peroxiredoxins are responsible for the reduction of 90% of intracellular H_2_O_2_. On the other hand, peroxiredoxins can be easily inactivated by H_2_O_2_, disabling peroxidase activity and therefore limiting their ability to act as antioxidant, particularly in an oxidative environment like inflammation and intermittent hypoxia [52]. Notably, enhancement of GSH levels was described in hypoxic intracellular environment [53, 54]. Glutathione (GSH) is considered to be the major thiol-disulfide redox buffer of the cell. On average, the GSH intracellular concentration is 0.5–10 mM [55]. This is far higher than most redox active compounds making GSH an important intracellular antioxidant and redox potential regulator that plays a vital role in drug detoxification and cellular protection from damage by free radicals, peroxides, and toxins [56]. Given the range of critical cellular functions involving GSH, it has long been considered that the modulation of intracellular GSH levels would be of great clinical benefits. Enhancement of GSH levels for cytoprotection is available by the administration of its precursor N-Acetyl cysteine, since direct administration of reduced GSH has physical and chemical limitations [56, 57]. Contrastingly, these cytoprotective effects of GSH and its associated enzymes in many types of cancer lead to an increased tumor cell survival and chemotherapy drug resistance [58].

#### 4.2.1. Glutathione and Chemoresistance

Preclinical studies of chemosensitization through antioxidant modulation have been reported in different tumor cells [44, 58–60]. However, chemoresistance is a complex system with multiple and heterogeneous mechanisms of action which are orchestrated not only by the tumor microenvironments but also by the biology of the tumor [61, 62]. Although most of the chemotherapeutic drugs are prooxidants, not all the cancer cell death induction pathways are ROS-dependent [43]. Nevertheless, chemoresistance is not caused by a single factor but rather contributed by combinations of many drug-resistant factors such as (1) reduced intracellular drug accumulation which may result from changes in drug transportation (increased efflux and decreased influx of anticancer drugs) and/or enhancement of detoxification activity; (2) increased DNA repair involving increased nucleotide excision repair, interstrand crosslink repair, or loss mismatch repair; (3) changes in the apoptotic cell death pathways; and (4) intracellular elevated antioxidant levels [63]. Among the antioxidants involved in the maintenance of intracellular redox balance, a main role is played by glutathione (GSH) [57]. GSH and its related enzymes participate not only in the antioxidant defense systems, but also in some drug-resistance metabolic processes such as detoxification and efflux of xenobiotics and blockage of the apoptosis tumor cell death pathway [58]. GSH is a major contributing factor to drug resistance by interacting with chemotherapeutic drugs such as cisplatin and trisenox [64, 65]. In fact, there are clinical evidences supporting a role of the GSH system in overcoming drug resistance and/or toxicity in solid tumors (e.g., lung cancers and bladder) treatments outcome [60, 63]. Therefore, much effort has been directed at depleting cellular GSH levels in order to sensitize tumor cells to the cytotoxic effects of anticancer drugs. The use of buthionine sulfoximine (BSO), an inhibitor of GSH synthesis [66], was performed in clinical trials [67–70]. However, the approach was limited by BSO availability and lack of selectivity of this drug for tumor versus normal cells [56]. But it is notable that GSH plays an important role in drug resistance and its depletion demonstrated to be effective in the sensitization of different types of cancer patients to cytotoxic chemotherapy [67–70]. Another alternative in progress is the development and optimization of GSH analogues that inhibit the enzyme glutathione-S-transferase (GST) responsible for the detoxification overcoming, therefore, chemoresistance [56, 71]. Among the GSH analogues developed, one (TLK 286), which is in clinical trial phase 3 settings for non-small-cell lung and ovarian cancer, appears to sensitize these tumors to cytotoxic chemotherapies [56]. However, the lack of tumor specificity is still a potential problem.

### 4.3. Antioxidant and Possible Clinical Benefits

**Figure 5:**
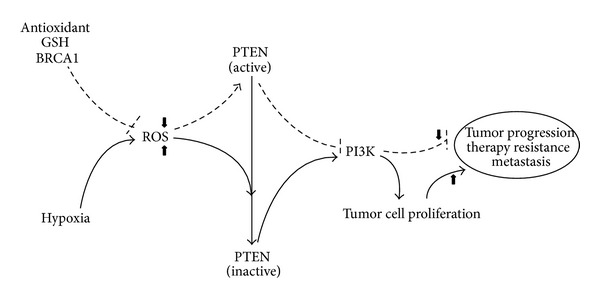
Possible clinical benefits of antioxidant in tumor progression.

Altogether, it should be recognized that understanding the redox biochemistry differences between normal and cancer cells is essential for the design and development of strategies to overcome oxidative damage or prooxidant chemoresistance [61, 62]. Even within a specific cancer type, the malignant cell populations are heterogeneous and intracellular oxidative levels may change as the disease progress [61]. Consequently, studying intra- and intertumor heterogeneous distribution of antioxidants levels may be an important factor to overcome tumor progression. Additionally, it is known that alterations in cellular redox metabolism play a crucial role in the activation or loss of tumor suppressor proteins activities such as breast cancer susceptibility gene breast cancer 1 (BRCA1) and phosphatase and tensin homolog deleted on chromosome 10 (PTEN) [3, 72–78]. The BRCA1 is an oncosuppressor gene with a relatively broad cellular role such as DNA repair (including nucleotide excision repair, NER, and double-strand break repair, DSBR), transcriptional regulation, and chromatin remodeling [79–83]. Notably, BRCA1 has also an antioxidant role in response to oxidative stress in which ROS cause DNA damage due to oxidation [73–75]. Loss or mutation of the BRCA1 gene was firstly described to be associated with increased risk of breast and ovarian cancers [80, 82–86]. Moreover, BRCA1 expression has been correlated with cancer aggressiveness and chemotherapy sensitivity in other solid tumors such as prostate [87–89], non-small-cell lung cancer [90–94], and pancreas [95, 96]. The superfamily of protein tyrosine phosphatase (PTP) enzymes functions in a coordinated pattern with protein tyrosine kinases to control the cellular regulatory signal processes such as cell growth, proliferation, and differentiation [97–100]. PTEN, a class 2 VH1-like (poxvirus vaccinia) DUSP (dual specificity phosphatase) [100] and likewise a member of phosphatase protein family, is modulated by ROS [98, 101]. The oxidation of the active site Cys by ROS abrogates PTEN catalytic activity and, thereby, switching on the phosphatidylinositol-3-kinase (PI3K) proliferation pathway [72, 75–77, 100, 101]. The tumor suppressor PTEN is one of the most frequently mutated genes in human cancer and is generally associated with advanced cancers and metastases [72]. A recent study reveals that PTEN loss activity is the most important alteration for cellular malignant transformation in mammary epithelial cells [102]. A recent study reveals that PTEN loss activity is a common event in breast cancers caused by BRCA1 deficiency [103] due to ROS enhancement. Recently, the modulation of other tumor suppressor genes was described to be ROS-dependent [78, 104, 105]. Therefore, the use of antioxidant might protect the biomarkers ROS-dependent tumorigenesis like BRCA1 and PTEN (Figure 5).

## 5. Conclusions

Enhancing the capacity of antioxidant in order to protect cells from redox-related changes or environmental toxins represents a persistent aim in the search for cytoprotective strategies against cancer. On the contrary, the strategy of depleting antioxidant is aimed at sensitizing cancer cells to chemotherapy, the so-called chemosensitization. In this context, it has been reported that antioxidant may be a determining factor for the sensitivity of some tumors to various chemotherapeutic agents. In particular, GSH and GSH enzyme-linked system are a relevant parameter for chemotherapy response, and it may be utilized as a useful biomarker for selecting tumors potentially responsive to chemotherapeutic regiments. The involvement of antioxidant in the carcinogenesis and in the drug resistance of tumor cell is clear, but further studies, aimed at understanding the antioxidant-driven molecular pathways and the biology of the tumor cells, are crucial to design new therapeutic strategies to fight cancer progression and overcome chemoresistance.

## Figures and Tables

**Table 1:** Endogenous antioxidants.

Endogenous antioxidants	Examples
Enzymes	Superoxide dismutase
	Catalase
	Peroxiredoxins
GSH enzyme-linked system	Glutathione peroxidase
	Glutathione S-transferase
	Glutathione reductase
Nonenzymes	Glutathione
	Cysteine
	Thioredoxin
Metal-binding proteins	Ferritin
	Metallothionein
	Ceruloplasmin

**Table 2:** Exogenous antioxidants.

Example of exogenous antioxidants		
Vitamin C	*⟶*	Ascorbate/ascorbic acid
Vitamin E	*⟶*	Tocopherols, tocotrienols
Carotenoids	*⟶*	*α*-carotene, *β*-carotene, lycopene
Polyphenols	*⟶*	Flavonols, flavanols, anthocyanins, isoflavones, phenolic acid
Trace elements	*⟶*	Selenium, copper, manganese, zinc
